# 液体活检在小细胞肺癌诊疗中的应用研究进展

**DOI:** 10.3779/j.issn.1009-3419.2022.101.22

**Published:** 2022-08-20

**Authors:** 晓敏 张, 梦圆 徐, 西川 李, 延军 苏

**Affiliations:** 1 300387 天津，天津师范大学生命科学学院，天津市动植物抗性重点实验室 Tianjin Key Laboratory of Animal and Plant Resistance, College of Life Sciences, Tianjin Normal University, Tianjin 300387, China; 2 300060 天津，天津医科大学肿瘤医院肺部肿瘤科，天津市肿瘤防治重点实验室，天津市肺癌诊治中心 Key Laboratory of Cancer Prevention and Therapy, Department of Lung Cancer, Tianjin Medical University Cancer Institute and Hospital, Tianjin 300060, China

**Keywords:** 肺肿瘤, 液体活检, 循环肿瘤DNA, 循环肿瘤细胞, 外泌体, Lung neoplasms, Liquid biopsy, Circulating tumour DNA, Circulating tumour cells, Exosomes

## Abstract

小细胞肺癌（small cell lung cancer, SCLC）是一种侵袭性强、死亡率高的恶性肿瘤，具有易转移、生长速度快、恶性程度高和侵袭性强等特点，患者预后一般较差。目前SCLC的临床诊断方法主要以组织活检为主，创伤大、周期长且成本高。近年来，液体活检以传统组织活检所不具备的无创性、全面性、实时性等优良特点而逐渐获得应用。液体活检的主要检测对象包括外周血中的循环肿瘤DNA（circulating tumor DNA, ctDNA）、循环肿瘤细胞（circulating tumors cells, CTCs）及外泌体等。将液体活检应用于SCLC的临床治疗，有助于提高临床医生对SCLC患者病情的详细诊断，以及对患者治疗反应的及时掌控和应对。

## Abstract (en)

Small cell lung cancer (SCLC) is a malignant tumor with strong invasiveness and high mortality. It has the characteristics of easy metastasis, fast growth, high degree of malignancy and strong invasiveness. The prognosis of patients is generally poor. The current clinical diagnosis of SCLC is mainly based on tissue biopsy, which is invasive, long cycle time and high cost. In recent years, liquid biopsy has been gradually applied because of its non-invasive, comprehensive and real-time characteristics that traditional tissue biopsy does not have. The main detection objects of liquid biopsy include circulating tumor DNA (ctDNA), circulating tumor cells (CTCs) and exosomes in peripheral blood. The application of liquid biopsy in the clinical treatment of SCLC will help clinicians to improve the detailed diagnosis of SCLC patients, as well as the timely control and response to the treatment response of patients.

肺癌是世界上发病率最高的恶性肿瘤之一，根据美国癌症协会统计，2020年全球肺癌发病率为14%，死亡率为18%^[1]^。肺癌按照组织病理学类型可分为小细胞肺癌（small cell lung cancer, SCLC）和非小细胞肺癌（non-small cell lung cancer, NSCLC），其中SCLC约占肺癌总发病率的15%^[2, 3]^。目前，组织活检是临床上肿瘤患者确诊的主要手段之一；但因其具有创伤性，可能导致患者出现气胸、出血等并发症而限制了其应用^[4]^。此外，约25%的肺癌活检因获得的组织样本过少而影响患者确诊与病情评估，因此探索一种新的检测技术对于临床检测是很有必要的。液体活检是指从以血液为主的体液中获得可用于检测分析生物标记物来获得组织相关信息的一种实验技术^[5]^。液体活检在肿瘤诊断等方面具有巨大的潜力，检测对象主要包括循环肿瘤DNA（circulating tumor DNA, ctDNA）、循环肿瘤细胞（circulating tumors cells, CTCs）及外泌体等。本文将对液体活检在SCLC的早期筛查诊断、病情监测及预后评判等方面的应用进展进行综述。

## 1 SCLC的概述

SCLC是一种恶性程度极高的神经内分泌肿瘤，临床上将其分为局限期（limited disease-SCLC, LD-SCLC）和广泛期（extensive disease-SCLC, ED-SCLC）^[6]^。SCLC患者的平均2年总生存率低于10%，其中LD-SCLC患者的中位生存期（median survival time, MST）为15个月-20个月，ED-SCLC患者的MST为8个月-13个月，且超过2/3的SCLC患者确诊时已属于ED-SCLC^[7-9]^。SCLC对于以铂类药物为基础的初次化疗较敏感，但病情易复发且复发后易对原化疗药产生获得性耐药^[10]^，预后极差。

SCLC细胞的形态学特征为卵圆形或梭形细胞、胞质少、细胞边界不清以及核仁不明显但核质明显等。肿瘤呈高增殖、高凋亡、高坏死，组织中Ki-67的阳性染色率为50%-100%^[10]^。目前对于SCLC患者极少进行手术，治疗手段主要是以化疗、放疗和免疫治疗相结合为主^[11]^。临床确诊的检测手段以经皮肺穿刺或气管镜取样后的病理检测为主，实际应用中具有较多限制；而液体活检在SCLC的临床诊断中逐渐获得了人们的重视。

## 2 液体活检的主要检测对象

### 2.1 ctDNA

无细胞DNA（cell-free DNA, cfDNA）是指细胞经过衰老、凋亡、坏死和胞外分泌等方式释放到血液和其他液体中的细胞外DNA分子；来源于肿瘤细胞的cfDNA被称为ctDNA。ctDNA大小为90个-150个碱基，主要存在于血液循环系统中^[12, 13]^，主要来源包括死亡的肿瘤细胞、CTCs、外泌体等。ctDNA可能携带有特异性改变的DNA片段，这些改变包括体细胞点突变、杂合性丢失、基因融合、基因拷贝数变异和DNA甲基化等^[11]^。

ctDNA在总cfDNA中的含量极低，对于检测点突变、拷贝数畸变（copy-number aberration, CNA）、小片段插入或缺失是一种挑战^[14]^，因此对其检测技术有较高的要求。目前ctDNA的分析方法主要有以突变阻滞扩增系统PCR（amplification refractory mutation system PCR, ARMS-PCR）技术为代表的定量PCR、以微滴数字PCR（droplet digital PCR, ddPCR）为代表的数字PCR、基于下一代测序（next-generation sequencing, NGS）的高通量技术^[11, 14, 15]^。BEAMing（bead, emulsion, amplification, magnetic）技术和ddPCR技术可以检测特定的已知突变，与肿瘤组织获得的结果高度一致；通过NGS对CNA或点突变进行全基因组分析可以获得更完整的全基因组表征以及患者的特异性基因分型，以此评估肿瘤的异质性及跟踪治疗过程中的基因改变。

### 2.2 CTCs

CTCs是始于原发或转移部位，进入循环系统后随之扩散到远处的肿瘤细胞。CTCs的半衰期较短，存在上皮细胞和间充质细胞标记物的杂交表型能够逃脱免疫细胞的“追踪”，有利于存活，能够随着血液循环系统到达远处^[11]^。CTCs在有核细胞中占有量极少，血液中只含有0.000, 000, 1%的CTCs，在外周血所有有核细胞（1个/mL-10个/mL）中只占0.001%左右^[16]^。

临床上对CTCs的检测主要包括分离、富集、再分析等过程，由于CTCs在血液中含量过低，无法通过常规外周血涂片的方法进行识别检测，因此富集在CTCs分析过程中十分重要。CTCs富集的物理方法包括：按尺寸过滤、使用介电性质或通过Ficoll密度梯度离心、通过免疫标志物阳性进行富集、使用抗体标记的微柱进行富集等，近几年来，使用白细胞共同抗原（leukocyte common antigen, LCA）CD45抗体耗尽白细胞进行富集是一种新兴的技术，此技术更加有利于从全血中分离、分析CTCs^[17]^。目前，CELLSEARCH^®^是唯一被美国食品药品监督管理局批准并认可的检测CTCs的标准技术，该技术使用免疫磁性捕获细胞表面表达的上皮细胞黏附分子（epithelial cell adhesion molecule, EpCAM），该系统中定义CTCs为有核的DAPI阳性、细胞角蛋白（cytokeratin, CK）阳性、CD45阴性细胞^[16]^。此外，检测CTCs的方法还有流式细胞检测术、逆转录PCR技术、荧光原位杂交技术、CTC芯片技术及膜滤过分离肿瘤技术（isolation by size of epithelial tumor cell, ISET）等^[15]^。

### 2.3 外泌体

外泌体是具有脂质双层膜的小细胞外囊泡，直径30 nm-100 nm，存在于包括血液、脑脊液、尿液在内的生物流体中^[18]^，外泌体中存在蛋白质、脂质、DNA和多种RNA（microRNA、mRNA、lnRNA、circRNA）分子，能够通过自分泌、旁分泌或内分泌途径在细胞与微环境之间传递信息^[19]^，参与血管生成、细胞增殖和迁移、炎症反应、免疫抑制、肿瘤免疫逃逸等过程^[15]^，并促进肿瘤的发生与发展。

外泌体的提取方法主要有超滤离心法、磁珠免疫法、密度梯度离心法、PEG-base沉淀法、色谱法等，其鉴定方法包括流式细胞术、实时荧光定量PCR、免疫胶体金标记技术和测序等^[15]^。

## 3 液体活检在SCLC的早期筛查诊断、病情监控及预后评估中的应用

### 3.1 液体活检在SCLC早期筛查诊断中的应用

SCLC易发生早期扩散，因此SCLC的早期筛查对于其治疗尤为重要。Fernandez-Cuesta等^[20]^研究了使用cfDNA样本检测SCLC患者*TP53*突变的可能性，研究结果显示，在49%具有不同肿瘤分期的SCLC患者的ctDNA中存在*TP53*突变；更重要的是按病程阶段对51例SCLC病例进行分组后，在35.7%的早期SCLC患者ctDNA中检测到了*TP53*突变。Nong等^[21]^分析了22例SCLC患者在治疗前和治疗中不同时间点的cfDNA样本，结果显示，基线检测时91%和67%的患者中分别存在*TP53*、*RB1*突变；对8例患者的血液及组织样本进行分析后，显示两者突变的一致性为94%，表明cfDNA测序是检测SCLC患者体细胞突变的敏感工具。以上研究提示对SCLC患者体内的ctDNA中TP53进行检测可作为早期筛查的手段之一。

Naito等^[22]^在对35例SCLC患者进行检测时发现，32%的SCLC患者在治疗前每7.5 mL血液中存在CTCs的数量 > 2个，此阳性率明显高于其他肿瘤。许多研究表明LD-SCLC与ED-SCLC患者之间CTCs数量存在差异。Hiltermann等^[23]^的研究结果显示LD-SCLC患者中检测到的CTCs水平要低于ED-SCLC患者；Aggarwal等^[24]^同样证明了ED-SCLC患者的CTCs水平要高于LD-SCLC患者。此外，Carter等^[25]^分析了SCLC患者血液CTCs中的CNA，并据此将SCLC患者分为化疗敏感型和化疗难治型。以上研究证明了对SCLC患者体内的CTCs进行检测可作为早期筛查和治疗方案选择的依据之一。

外泌体在癌症患者中的水平通常高于健康者^[26]^，疾病会促进外泌体的循环，其膜组成分及对分子载体的表征是生物标志物的重要来源^[18]^。Sandfeld-Paulsen等^[27]^在应用细胞外囊泡阵列对外泌体进行表征后，发现SCLC血浆中CD151标志物的数量显著上调。Pedersen等^[28]^利用蛋白质组学技术对24例健康者和24例SCLC患者外泌体与囊泡中获得的蛋白质进行定量与统计分析，发现在健康者与SCLC患者中存在几种差异表达的蛋白，这一差异表明外泌体中可能包含具有SCLC潜在诊断标志物的特定蛋白质。由此可见，对于外泌体的分析有助于SCLC患者的早期筛查和诊断。

### 3.2 液体活检在监控SCLC病情变化中的应用

在SCLC的临床治疗中，患者的基因组处于动态变化之中，不同患者对于使用药物及放化疗的反应也不相同^[29]^。目前临床上一般通过血清标志物、影像学和支气管镜检查等技术对患者的疾病进展情况进行监测^[30]^；但存在准确率低、检测范围小且费用昂贵等问题。另外，临床上支气管镜取样本后进行的传统组织活检获得的是肿瘤的局部照片，单个活检样本无法捕捉到肿瘤完整的基因组图谱。因此，这两类检测技术对于监控病情的变化均具有一定的局限性；而液体活检可以在患者治疗过程中获取患者体内肿瘤在分子水平上发生的动态变化。

Du等^[31]^对24例SCLC患者的cfDNA及对应基因组DNA进行靶向测序，观察到SCLC患者中存在着广泛的体细胞拷贝数改变和突变，基因突变指数与无进展生存期（progression‐free survival, PFS）、总生存期（overall survival, OS）显著相关，该研究证明了基于cfDNA的液体活检对于SCLC患者的临床反应监测具有潜在作用。随后的一项研究^[32]^表明，基于cfDNA的全基因组拷贝数是监测患者病情变化的有效方法，并且靶向cfDNA测序可识别50%以上SCLC患者的潜在治疗靶点。

Poroyko等^[33]^对SCLC和NSCLC患者血清外泌体中的miRNA进行测序，确定了17种miRNA在SCLC患者与NSCLC患者中差异表达；与NSCLC患者相比，接受化疗后的SCLC患者外泌体中的miRNA变化更加明显，证明外泌体miRNA可作为标志物显示SCLC患者对治疗的反应。

有多项研究^[22-24]^认为CTCs的计数变化与化疗反应基本无相关性，但在Ju等^[34]^的研究中，对14例SCLC患者化疗前后分别进行CTCs计数，确定CTCs计数的变化与治疗反应相关。CTCs计数变化是否能够准确反映SCLC患者治疗过程中病情的进展尚不确定，仍需要进一步研究。

### 3.3 液体活检在预测SCLC患者预后中的应用

SCLC患者治疗后复发率高是临床上难以攻克的问题，而有效预测患者的预后效果能够更好地保证患者的后续治疗。Thomas等^[35]^描述了1例*BCRA1*突变的SCLC患者，在II期实验中对该患者使用Olaparib和Durvalumab联合治疗，联合用药后患者病情得到很大缓解，患者ctDNA中的*BCRA1*基因突变频率也明显下降，此研究暗示可利用检测SCLC患者ctDNA中某一基因的突变频率来预测预后效果。Zhang等^[36]^对14例SCLC患者血浆ctDNA中变异等位基因频率（variant allele frequency, VAF）的变化水平和趋势进行评估，结果显示，血浆ctDNA中VAF的变化趋势可作为生物标志物评估化疗对SCLC患者和晚期SCLC患者的疗效，且ctDNA分析揭示的患者疾病进展情况比影像学报告更早。

多名研究者^[23, 37]^证明ED-SCLC的CTCs计数高于LD-SCLC患者的CTCs计数，SCLC患者化疗后的CTCs计数是预测OS的最强预测因素，CTCs较高的患者生存期大多数低于初始CTCs数量低的患者。Naito等^[22]^对51例SCLC患者的不同病程阶段（包括基线、化疗后、复发阶段）抽取血液样本，使用CellSearch System对采集的血液样本进行分层分析，最终结果显示，SCLC患者中CTCs的维持在较高的水平，CTCs水平越高，患者生存率越差。总体而言，以上研究表明液体活检将在SCLC患者的预后管理中成为关键因素。

### 3.4 液体活检在SCLC其他方面的作用

随着研究的进一步深入，液体活检在其他方面的应用也逐渐得到重视。CTCs的单细胞测序可以提供SCLC突变图谱的相关信息。Ni等^[38]^研究证明CNA对CTCs的单细胞测序具有鉴别SCLC与肺腺癌的潜力。Li等^[39]^通过利用血小板靶向慢病毒转基因法使血小板能够特异性表达肿瘤坏死因子相关的凋亡诱导配体（tumor necrosis factor-related apoptosis-inducing ligand, TRAIL），与CTCs进行靶向结合，能够减缓CTCs的转移。另有研究者^[40]^通过基因组学和转录组学表征CTCs的分子图谱，研究肿瘤的异质性，构建CTCs的异种移植模型，为探索肿瘤对化疗药物耐药性的机制提供补充信息，有望用于为SCLC患者提供个性化治疗。

外泌体是人体转运RNA的内源性系统，已被证实具有调节免疫反应的功能，可以作为肿瘤和感染的无细胞疫苗，也具有成为治疗性RNA递送系统的潜在可能^[41]^。Mao等^[42]^首次证明了外泌体miR-141/KIF12途径在SCLC血管生成中的作用，为SCLC患者的治疗提供了新的潜在靶点。Dou等^[43]^的研究证明抗程序性死亡配体-1（programmed death ligand-1, PD-L1）阻断抗体显著逆转了外泌体介导的CD8^+^ T细胞活化的抑制，该结果表明，含有PD-L1的外泌体可以增强T细胞介导的针对SCLC的免疫治疗。

## 4 小结与展望

液体活检在实时性、准确性、无创性等方面具有极大的优势。液体活检的无创性有望缩短SCLC患者的治疗恢复期，通过ctDNA、CTCs以及外泌体等检测手段获取组织活检无法获得的动态信息，能够实时监测患者治疗后的病情变化以及预测患者的预后状态，同时也可能在SCLC靶点追踪、疫苗研制等方面发挥重要作用。

另外，需要指出的是，目前液体活检在SCLC的临床应用中仍然具有一定的局限性。一方面，虽然ctDNA的灵敏性高、可靠性强，但是CTCs、外泌体在患者体内含量较少，这对检测中的富集技术和灵敏度提出了很高的要求；另一方面，为了达到灵敏度、准确度方面的要求，检测成本会随之升高，可能影响其在临床的广泛应用。除此之外，CTCs在化疗敏感性以及治疗反应方面仍需要进一步研究证实。临床上仍不能将液体活检获得的结果作为唯一的参照，而是应当将液体活检数据与组织活检、影像学检测及其他检测技术结合起来用于SCLC患者的临床治疗。
